# Supplementary material for: Ontogeny, species identity, and environment dominate microbiome dynamics in wild populations of kissing bugs (Triatominae)
Source: Microbiome. 2020 Oct 11;8:146. doi: 10.1186/s40168-020-00921-x (PMC7549230; doi:10.1186/s40168-020-00921-x)
Supplement: Supplementary file 3 — Additional File 2: Methodological Supplement for 18S rRNA gene blocking primer design and validation [file 40168_2020_921_MOESM2_ESM.pdf]

Ontogeny, species identity and environment dominate microbiome dynamics in wild populations of kissing bugs (Triatominae)

Joel James Brown [1, 2], Sonia M. Rodríguez-Ruano [+ 1], Anbu Poosakkannu [1], Giampiero Batani [1], Justin O. Schmidt [3], Walter Roachell [4], Jan Zima Jr.[1], Václav Hypša [1] & Eva Nováková [1,5*]

[1] University of South Bohemia, Faculty of Science, Ceske Budejovice, Czech Republic
[2] Biology Centre of the Czech Academy of Sciences, Institute of Entomology, Ceske Budejovice, Czech Republic
[3] Southwestern Biological Institute, Tucson, Arizona, USA
[4] US Army Public Health Command-Central, JBSA Fort Sam Houston, Texas, USA
[5] Biology Centre of the Czech Academy of Sciences, Institute of Parasitology, Ceske Budejovice, Czech Republic

Additional File 2: Methodological Supplement for 18S rRNA gene blocking primer design and validation

Background

Since 2010, two general primer pairs 515F/806R [1] and 515F/926R [2] that were introduced by the Earth Microbiome Project (EMP [3]) have been broadly used in numerous studies (cumulative number of citations according to Web of Science equals 3334 as of August 14, 2020). Both primer combinations have highly comprehensive coverage that spans sequences of small ribosomal subunits of Bacteria, Archaea but also Eukaryota; i.e. 16S rRNA and 18S rRNA gene amplicons. The number of 18S rRNA reads amplified with 515F/806R was previously evaluated from mosquito samples, averaging 4% for single individuals and pooled templates (calculated from the average read numbers [4]). For the 515F/926R primer pair [2] the authors note even lower specificity towards the bacterial 16S rRNA gene. On average, they retrieved 17% of 18S rRNA gene amplicons from plankton samples. In principle, there are two main reasons why the general amplification properties and potential biases in amplicon analyses with the EMP primer pairs have been broadly overlooked. The first is solely methodological: the data are processed with various analytical pipelines that remove non-overlapping paired-end reads. 18S rRNA reads are approximately 200bp longer and thus discarded and not analysed further. The second reason originates in biological properties of analysed samples, i.e. different proportions of bacterial and eukaryotic DNA. Thus, while some studies can benefit from the comprehensive coverage of EMP primer pairs (e.g. using 18S rRNA reads for the host molecular taxonomy [4]), these might pose a major drawback for microbial analyses of templates with a low proportion of targeted bacterial DNA. Our initial trial for microbiome analyses of hematophagous kissing bugs (Triatominae) with 515F/926R primers failed due to preferential amplification of 18S rRNA sequences, reaching up to 100% of retrieved reads in some samples (as illustrated here in Figure 3C). We assumed that such a bias stems from a high eukaryotic content of our samples, consisting of Triatominae gDNA, prey gDNA from the blood meal, and gDNA of eukaryotic parasites associated with kissing bugs (e.g. *Trypanosoma cruzi*, *Trypanosoma rangeli*, *Hepatozoon* sp.). This may be overcome by using an 18S rRNA blocking primer.

Methods

Design of 18S rRNA gene blocking primer and initial PCR evaluation

While we have primarily designed the blocking primer (designated here as 926X) to lower the numbers of 18S rRNA amplicons retrieved for various *Triatoma* species, its annealing site is conserved in representatives of 23 Insecta orders, a human and a mouse (a single nucleotide mismatch was found for Thysanoptera, Psocoptera and Strepsiptera; Figure 1 and Additional File 1). The eight bp at the 5' end of the blocking primer 926X (5' GTGCCCTT**CCGTCAATTCCT**-C3 3') specifically match the 18S rRNA gene sequences, while the last 12 bp partially overlap with the 926R (5' **CCGYCAATTYMT**TTRAGTTT 3') annealing site. We used a 3' C3 spacer CPG modification (available from most suppliers of custom oligos) that prevents elongation during PCR and does influence annealing properties [5].

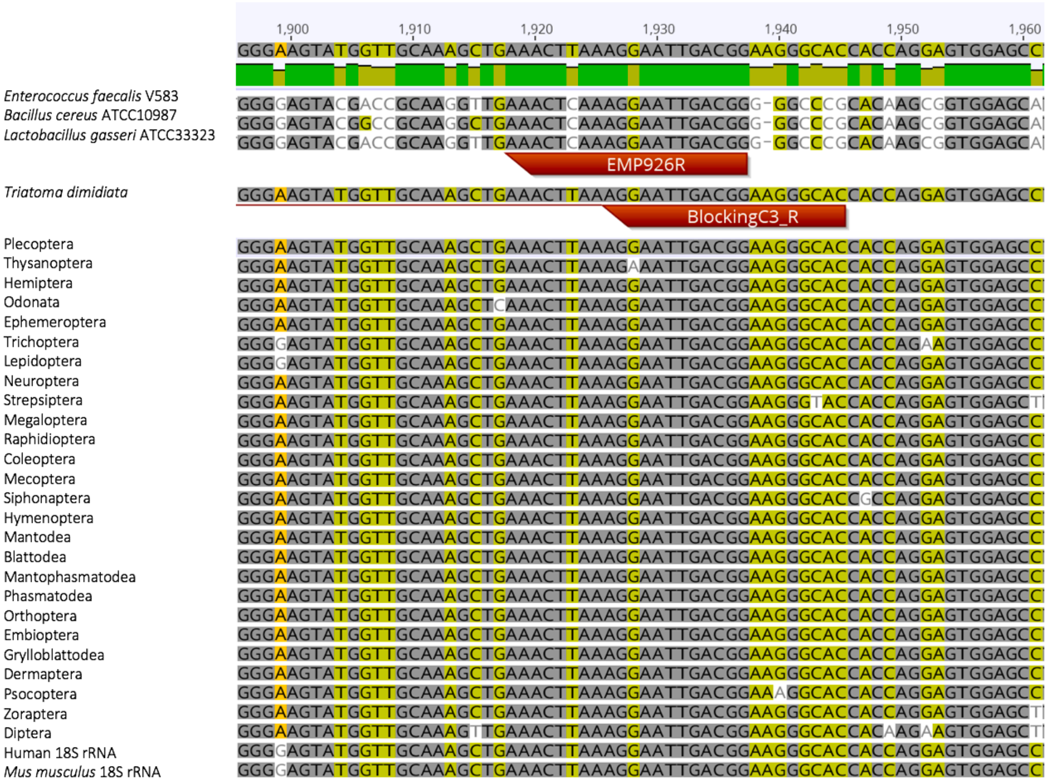

Figure 1. Illustration of 18S rRNA sequences alignment composed of 26 insect orders along with human, mouse, and *Triatoma dimidiata* sequences showing the conservative annealing site of the 926X blocking primer. Three 16S rRNA sequences were included to illustrate the mutual position of 926R amplification and 926X blocking primers.

The performance of the 926X blocking primer was initially evaluated by a simple PCR assay and gel electrophoresis using four DNA templates (A and B not producing a detectable 16S rRNA PCR product with 515F/926R primers and C and D producing a faint band (Figure 2). The blocking primer was added to 50 µL reaction with Q5 High-Fidelity 2X Master Mix in tenfold higher concentration compared to that of 515F/926R primers (final concentrations of 5 µM and 0.5 µM, respectively). PCR conditions as recommended by the EMP 16S Illumina Amplicon Protocol (https://press.igsb.anl.gov/earthmicrobiome/protocols-and-standards/16s/) were followed.

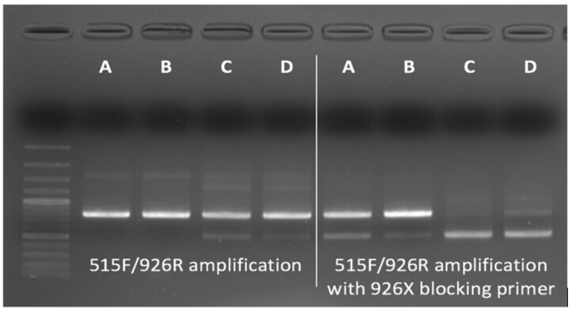

Figure 2. Gel electrophoresis of PCR products amplified from four different Triatominae DNA templates (A-D) with 515F/926R primers (rows 2-5) and with 515F/926R in combination with the novel 926X blocking primer (rows 6-9). Upper bands represent 18S rRNA products (app. 740 bp), lower bands are 16S rRNA products (app. 470 bp). The ladder used is Gene Ruler 100 bp Plus (Thermofisher Scientific).

926X evaluation using amplicon sequencing

The blocker performance was further evaluated using two 16S rRNA gene libraries constructed from the same 47 Triatominae DNA templates (extraction protocol is described in 2.2 section of the main text). While the "*regular*"16S rRNA gene library was amplified solely with double barcoded 515F/926R primer pair of the EMP protocol [2], the "*blocked*" library also employed the novel 18S rRNA gene blocking primer (metadata are provided in Additional File 1). Each library contained two negative controls (PCR water template and blank extraction control) and a single positive control from a previously sequenced *R. prolixus* adult isolated from our laboratory colony [6]. The PCR products were amplified as described above, cleaned with AMPure XP (Beckman Coulter) magnetic beads, pooled and additionally purified using Pippin Prep (Sage science) (see 2.5 section of the main text). Amplicons subjected to this trial were sequenced with 300 cycle Nano V2 chemistry in a multiplexed low output run of Illumina MiSeq. Altogether, the run contained ribosomal amplicon pools retrieved from 192 samples intended for other studies.

Data processing and analyses

The raw data comprised 897, 897 high quality paired reads. Since Illumina technology cannot currently read through the full length of 18S rRNA amplicons retrieved with 515F/926R primers (app. 740bp), we opted for stitching R1 and R2 reads using *fastq_join* script of USEARCH v9.2.64 [7].

Reads were demultiplexed, joined and quality filtered, and the dataset was clustered as described in 2.6 section of the main text. Taxonomy was assigned to the representative sequences using the BLAST algorithm [8] against the SILVA 132 SSU database [9].

Results and Conclusion

Out of 47 template pairs (amplified with and without the novel 926X blocking primer), 2 pairs produced extremely low amounts of data (29 and 62 total reads) and were not further analysed. The negative controls of the "*regular*" library contained 44 and 8 reads, while those of the "*blocked*" library comprised 6 and 104 reads (all the reads were assigned to 8 OTUs representing *Chrysobacterium*, two *Geobacillus* OTUs, two Thermaceae OTUs, *Deinococcus*, *Bacillus* OTU38 and *Sphingomanas*). The single positive control in the "*blocked*" library with 3251 total reads comprised an expected profile of previously sequenced *R. prolixus* [6], i.e. 80.9% *Enteroccoccus*, 6.9% *Bacillus* OTU15, 2.1% *Arsenophonus* and 10.1% of non-bacterial reads. Recalculated as 90%, 7.6%, and 2.4% of the bacterial reads, the profile mirrors those of the positive controls used in our main experiment (section 3.1 of the main text).

The read number retrieved per sample from the "*regular*" library was 4505 (±126). The "*blocked*" library produced notably lower numbers or reads per sample (873 ±195), Figure 3A. However, the proportion of 16S rRNA reads was extremely low in the "*regular*" library (on average 2% ±2) compared to 29% ±23% in the "*blocked*" library; Figure 3). On average, implementing the 926X blocking primer increased the bacterial read yield by 27%. In other words, we present 10% or higher improvement of 16S rRNA gene amplification in over 73% of our samples (Figure 3).

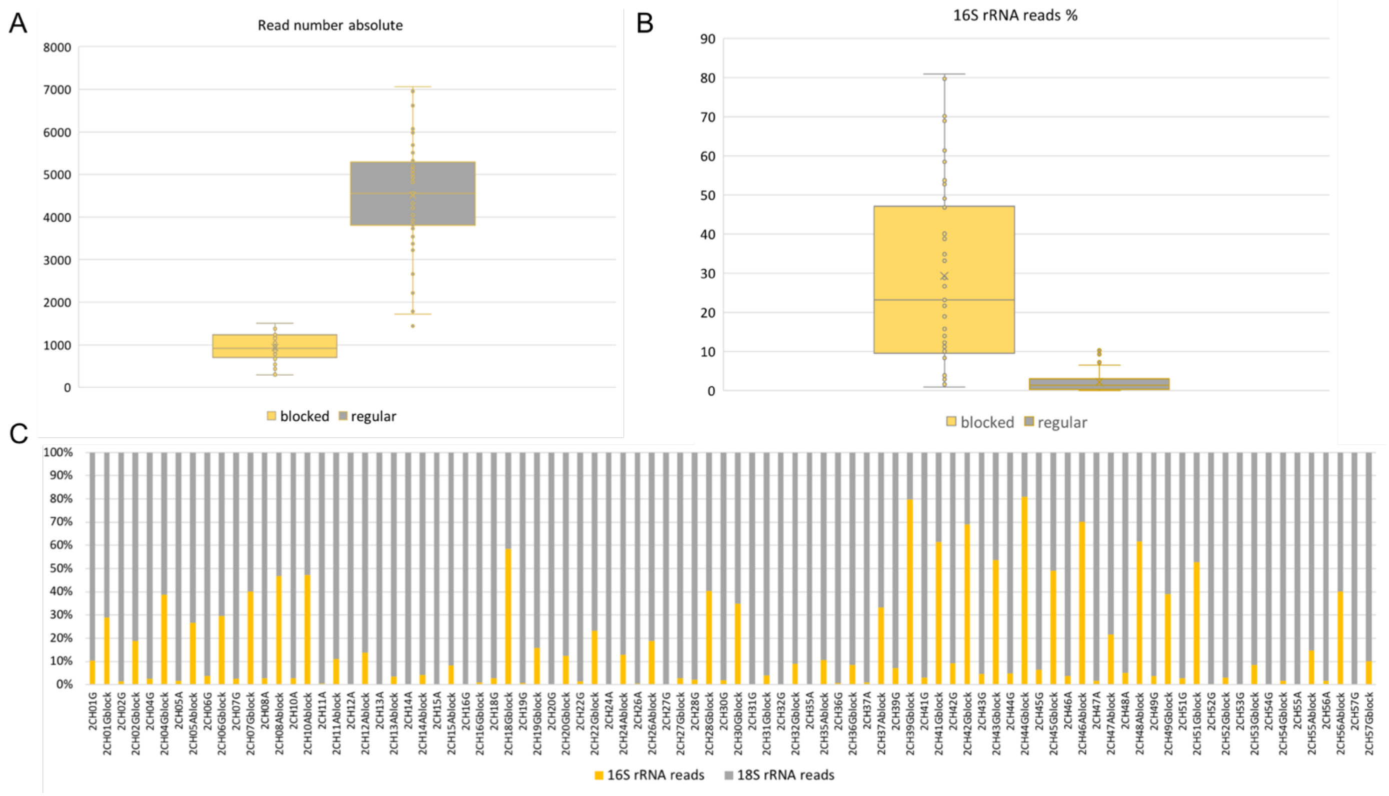

Figure 3. Improvement of 16S rRNA gene amplification using 926X blocking primer. Absolute read number retrieved after data processing for the samples from the "*regular*" library and "*blocked*" library (**A**). Proportion of 16S rRNA reads in the "*regular*" library and "*blocked*" library (**B**). Proportional values of 16S rRNA and 18S rRNA reads retrieved for each of the samples in the "*regular*" library and "*blocked*" library (organized in paired order from left to right; **C**).

We assume that the difference between the number of reads retrieved here from the "*regular*" and "*blocked*" library stems from non-equimolar proportion between the two pooled libraries rather than the 926X blocking primer directly reducing read numbers. We support our assumption with the results presented in the main body of this study (see 3.1 section of the main text). There, we have implemented the 926X blocking primer in a highly multiplexed library of 480 samples and did not experience any particular reduction of data retrieved with a regular output mode of Illumina MiSeq (V3 chemistry 600 cycles).

While our *in silico* prediction suggests potentially more general use of the 926X blocking primer, further validation with various templates, especially those containing a high proportion of eukaryotic DNA, should be performed. So far, we have tested its performance with positive results (enhancing 16S rRNA gene amplification) in our current projects with Anoplura, Diptera (Hippoboscidae), and Sternorrhyncha (Aleyrodidae); data not shown.

Data availability

All metadata for the samples used in this evaluation trial are provided in Additional File 1. Demultiplexed data have been deposited in ENA under following accession number: PRJNA657483

References:

1. Caporaso JG, Lauber CL, Walters WA, Berg-Lyons D, Lozupone CA, Turnbaugh PJ, et al. Global patterns of 16S rRNA diversity at a depth of millions of sequences per sample. Proc Natl Acad Sci. 2011;108 Supplement 1:4516–22.

2. Parada AE, Needham DM, Fuhrman JA. Every base matters: assessing small subunit rRNA primers for marine microbiomes with mock communities, time series and global field samples. Environ Microbiol. 2016;18:1403–14.

3. Gilbert JA, Meyer F, Jansson J, Gordon J, Pace N, Tiedje J, et al. The Earth Microbiome Project: Meeting report of the "1st EMP meeting on sample selection and acquisition" at Argonne National Laboratory October 6 th 2010. Report. BioMed Central; 2010. https://environmentalmicrobiome.biomedcentral.com/articles/10.4056/aigs.1443528. Accessed 15 Aug 2020.

4. Nováková E, Woodhams DC, Rodríguez-Ruano SM, Brucker RM, Leff JW, Maharaj A, et al. Mosquito microbiome dynamics, a background for prevalence and seasonality of West Nile virus. Front Microbiol. 2017;8:526.

5. Vestheim H, Jarman SN. Blocking primers to enhance PCR amplification of rare sequences in mixed samples – a case study on prey DNA in Antarctic krill stomachs. Front Zool. 2008;5:12.

6. Rodríguez-Ruano SM, Škochová V, Rego ROM, Schmidt JO, Roachell W, Hypša V, et al. Microbiomes of North American Triatominae: the grounds for Chagas disease epidemiology. Front Microbiol. 2018;9:1167.

7. Edgar R. Usearch. Lawrence Berkeley National Lab. (LBNL), Berkeley, CA (United States); 2010. https://www.osti.gov/sciencecinema/biblio/1137186. Accessed 4 Nov 2019.

8. Camacho C, Coulouris G, Avagyan V, Ma N, Papadopoulos J, Bealer K, et al. BLAST+: architecture and applications. BMC Bioinformatics. 2009;10:421.

9. Quast C, Pruesse E, Yilmaz P, Gerken J, Schweer T, Yarza P, et al. The SILVA ribosomal RNA gene database project: improved data processing and web-based tools. Nucleic Acids Res. 2013;41:D590–6.
