# Supplementary material for: Ontogeny, species identity, and environment dominate microbiome dynamics in wild populations of kissing bugs (Triatominae)
Source: Microbiome. 2020 Oct 11;8:146. doi: 10.1186/s40168-020-00921-x (PMC7549230; doi:10.1186/s40168-020-00921-x)
Supplement: Supplementary file 4 — Additional File 3: Results of ordination analyses based on 'basic' and 'decontam' datasets. [file 40168_2020_921_MOESM3_ESM.pdf]

Additional File 3: Results of ordination analyses based on '*basic'* and '*decontam'* datasets.

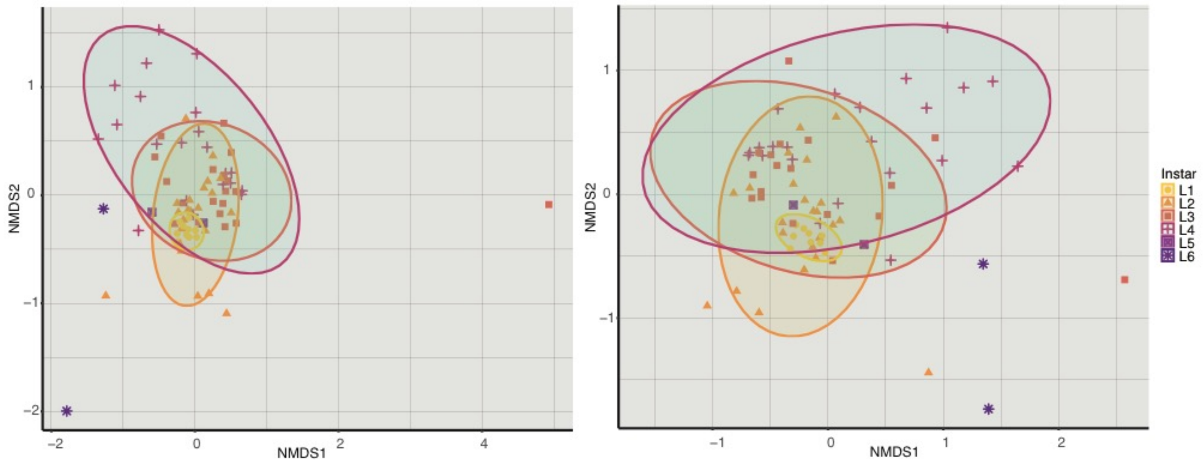

A (Supplementing Figure 1C): NMDS based on Bray-Curtis dissimilarity matrix calculated for *Triatoma rubida* ontogeny from the '*basic*' dataset (left) and '*decontam'* dataset (right). Each colour and shape correspond to a different instar; L6 stands for adults. Ellipses are statistically significant at the confidence interval of 0.05.

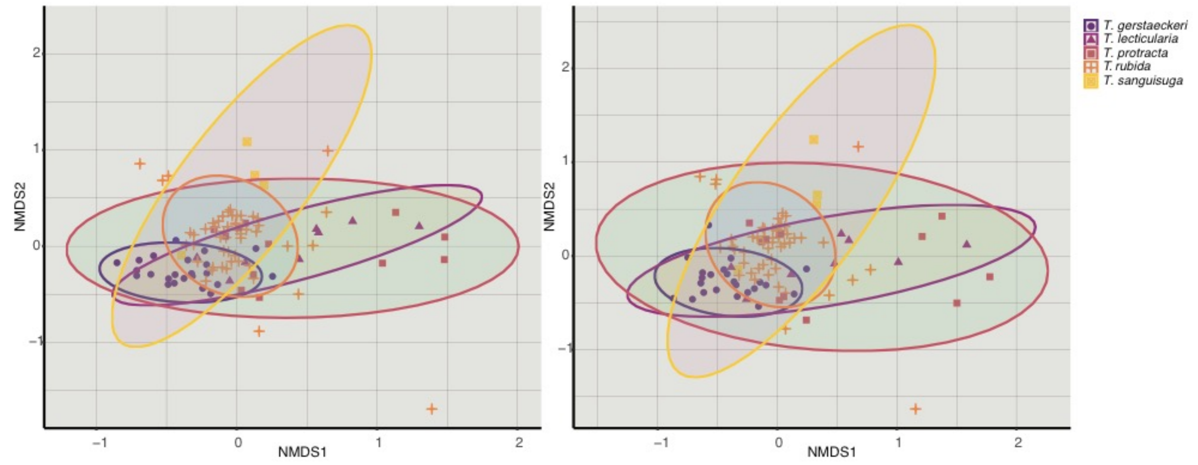

B (Supplementing Figure 3): Microbiome species-specific differences. NMDS ordination for the early instar dataset (L1-L3) of five *Triatoma* species based on Bray-Curtis dissimilarities calculated from from the '*basic*' dataset (left) and '*decontam'* dataset (right).

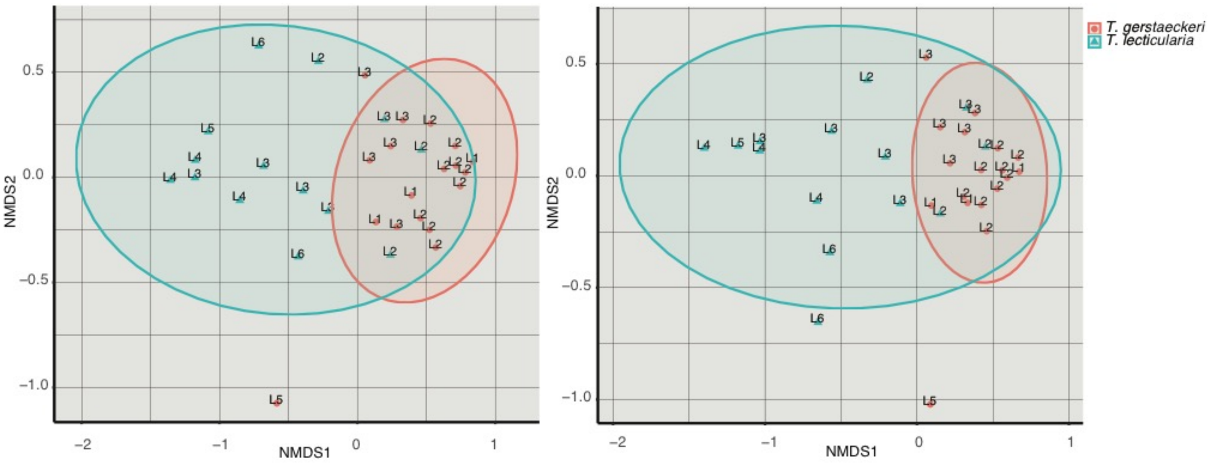

C (Supplementing Figure 4): Non-metric multidimensional scaling analysis for for *Triatoma gerstaeckeri* and *Triatoma lecticularia* from the same nest in Chaparral, TX; calculated from from the '*basic*' dataset (left) and '*decontam'* dataset (right).

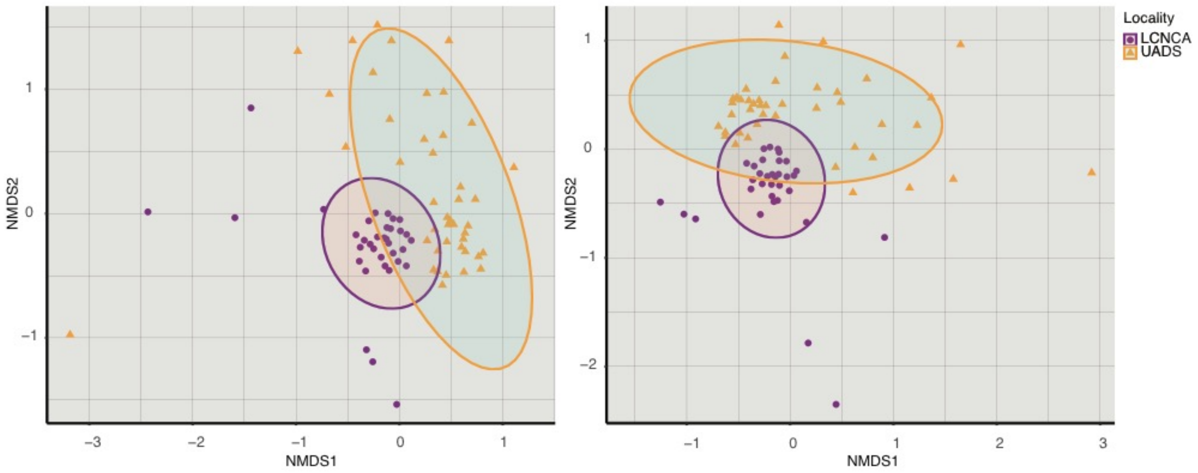

D (Supplementing Figure 6): Non-metric multidimensional scaling analysis for *T. rubida* collected from two locations in southern Arizona. LCNCA stands for Las Cienegas National Conservation Area, UADS for University of Arizona Desert Station; calculated from from the '*basic*' dataset (left) and '*decontam'* dataset (right).
