# Supplementary material for: Ontogeny, species identity, and environment dominate microbiome dynamics in wild populations of kissing bugs (Triatominae)
Source: Microbiome. 2020 Oct 11;8:146. doi: 10.1186/s40168-020-00921-x (PMC7549230; doi:10.1186/s40168-020-00921-x)
Supplement: Supplementary file 6 — Additional File 5: Microbiome ontogenetic shift in other Triatoma sp. [file 40168_2020_921_MOESM5_ESM.pdf]

Additional File 5: Microbiome ontogenetic shift in other *Triatoma sp.*

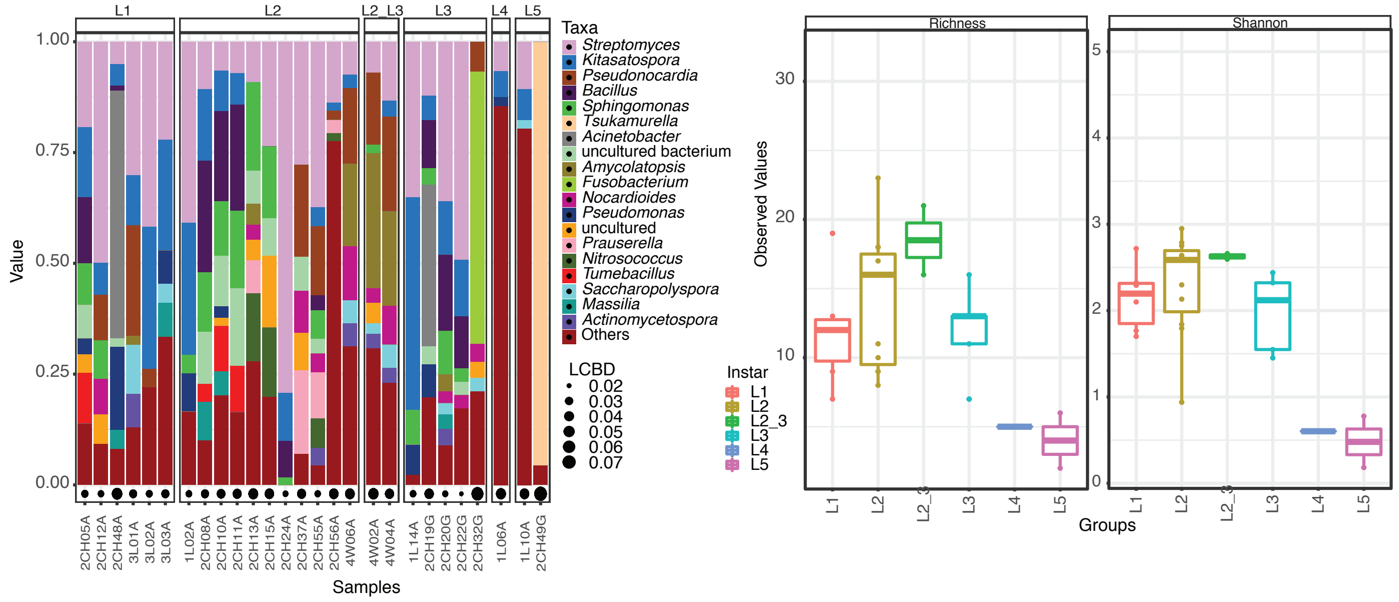

A (Supplementing Figure 1B): Microbiome profiles (top 20 bacterial genera) and diversity indices for the ontogenetic range of *Triatoma gerstaeckeri* based on the '*ultraclean*' dataset.

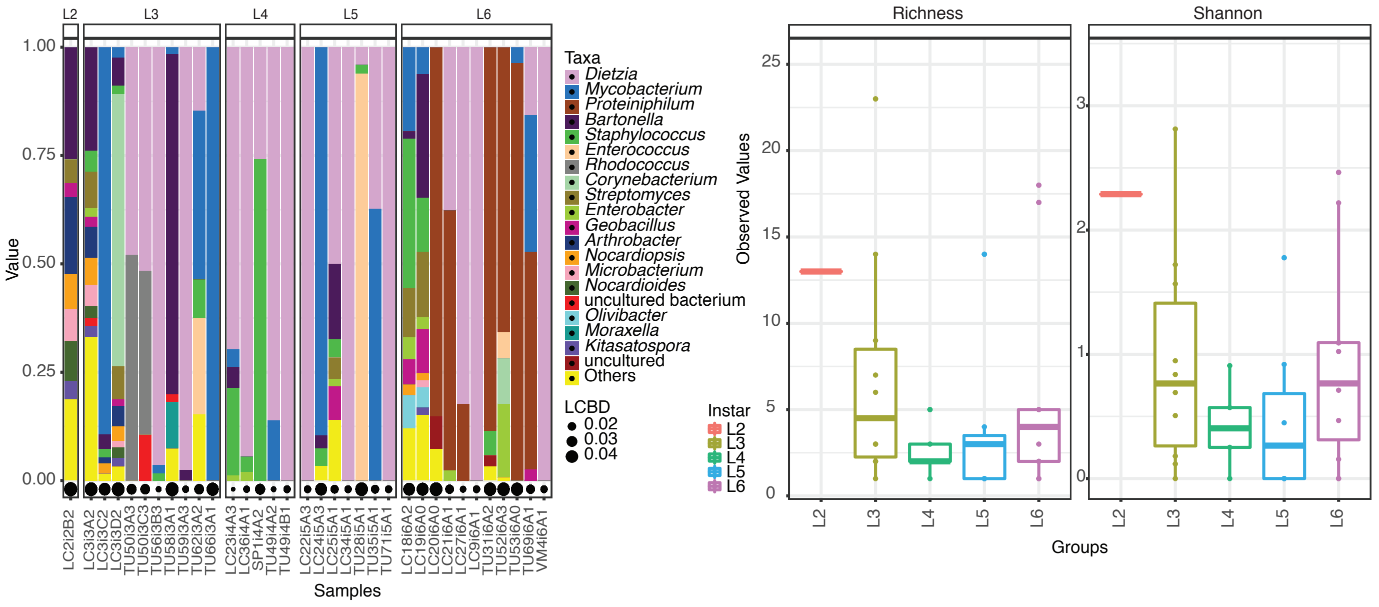

B (Supplementing Figure 1B): Microbiome profiles (top 20 bacterial genera) and diversity indices for the available ontogenetic range of *Triatoma protracta* based on the '*ultraclean*' dataset. Pink denotes *Dietzia* which displays a similar cumulative pattern to *T. rubida* discussed in the main manuscript.

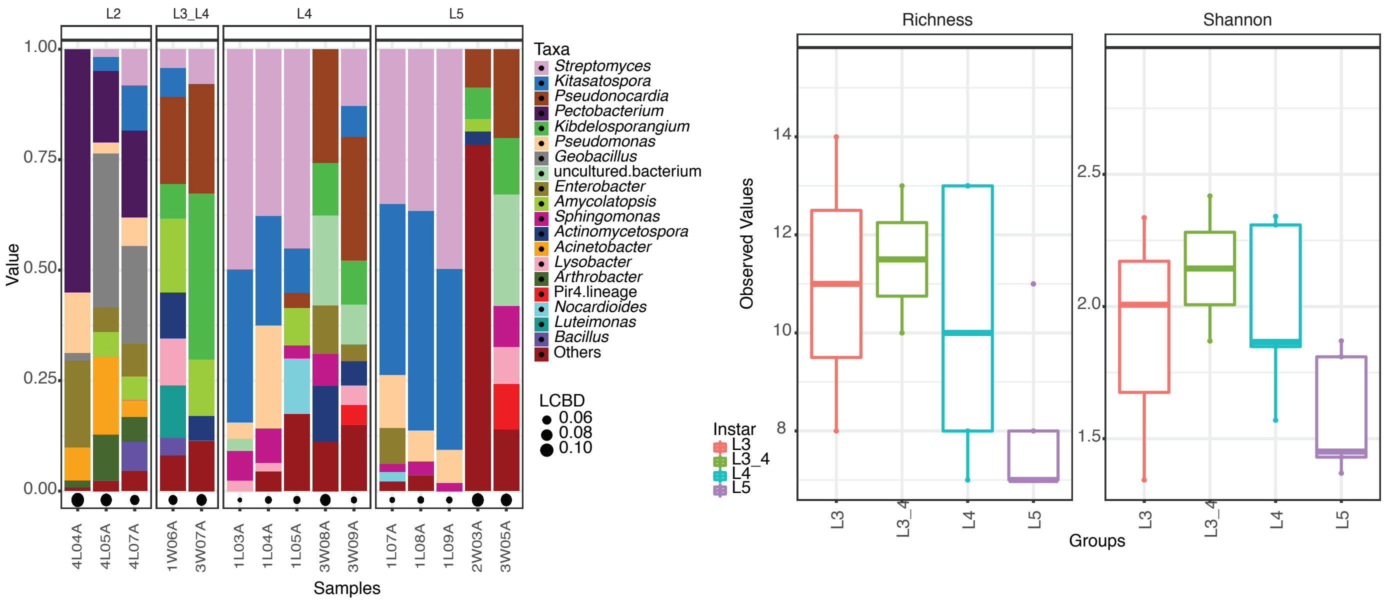

C (Supplementing Figure 1B): Microbiome profiles (top 20 bacterial genera) and diversity indices for the available ontogenetic range of *Triatoma sanguisuga* based on the '*ultraclean*' dataset.

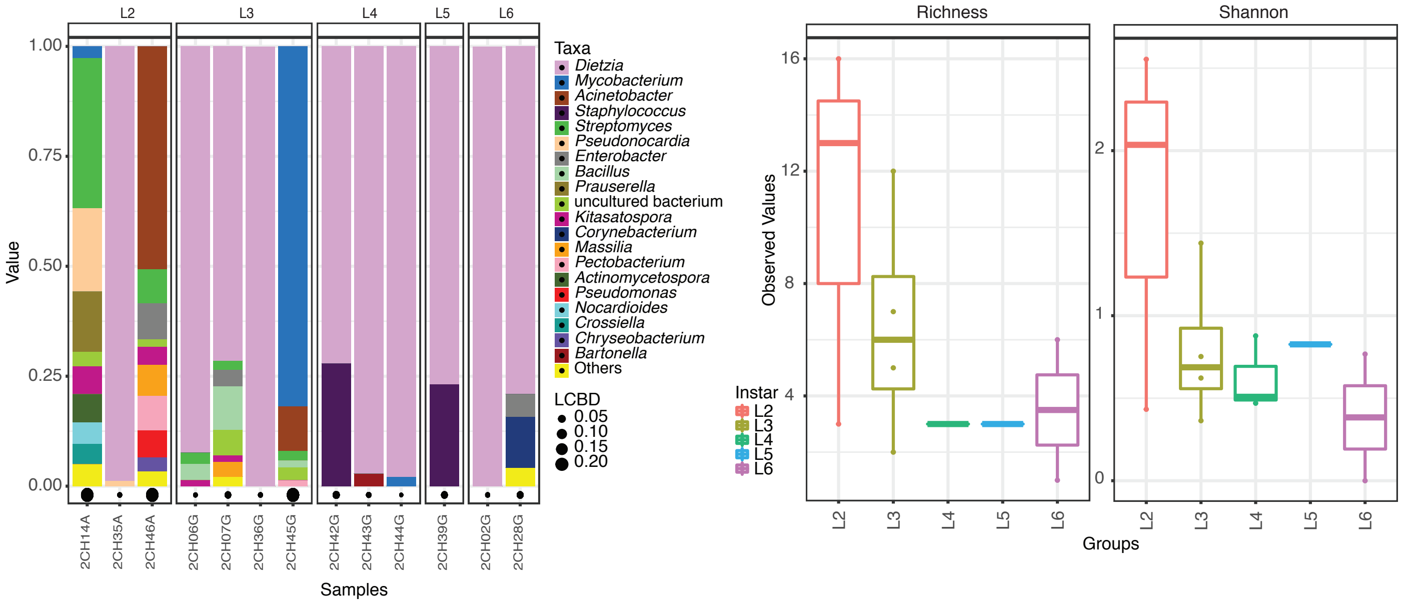

D (Supplementing Figure 1B): Microbiome profiles (top 20 bacterial genera) and diversity indices for the available ontogenetic range of *Triatoma lecticularia* based on the '*ultraclean*' dataset. *T. lecticularia* is the third (after *T. rubida* and *T. protracta*) of our study species to display a similar pattern of *Dietzia* over ontogenetic development.
