# Supplementary material for: Ontogeny, species identity, and environment dominate microbiome dynamics in wild populations of kissing bugs (Triatominae)
Source: Microbiome. 2020 Oct 11;8:146. doi: 10.1186/s40168-020-00921-x (PMC7549230; doi:10.1186/s40168-020-00921-x)
Supplement: Supplementary file 7 — Additional File 6: Significant difference in beta dispersion of the instar range groups (L1-L3 and L4-L6) calculated from the ultraclean dataset. [file 40168_2020_921_MOESM6_ESM.pdf]

	Df	Sum	Sq Mean	Sq F value	Pr(>F)
Groups	1	0.5625	0.56249	11.749	0.0007677 ***
Residuals	166	7.9475	0.04788		

Signif. codes: 0 '***' 0.001 '**' 0.01 '*' 0.05 '.' 0.1 ' ' 1

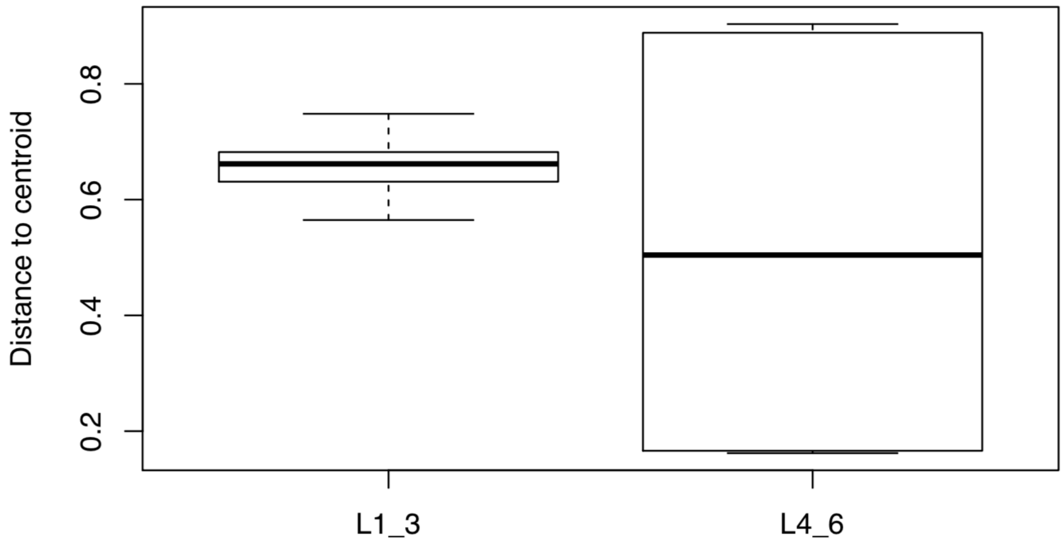

Additional File 6: Significant difference in beta dispersion of the instar range groups (L1-L3 and L4-L6) calculated from the *ultraclean* dataset. The beta dispersion analyses were performed with Jaccard index distance matrix using Vegan package (v2.5.6) in R software (v3.6.1).
