# Supplementary material for: Ontogeny, species identity, and environment dominate microbiome dynamics in wild populations of kissing bugs (Triatominae)
Source: Microbiome. 2020 Oct 11;8:146. doi: 10.1186/s40168-020-00921-x (PMC7549230; doi:10.1186/s40168-020-00921-x)
Supplement: Supplementary file 8 — Additional File 7: NMDS analyses of T. rubida microbiomes from early instar (L1-L3) individuals found in different N. albigula nests at UADS. [file 40168_2020_921_MOESM7_ESM.pdf]

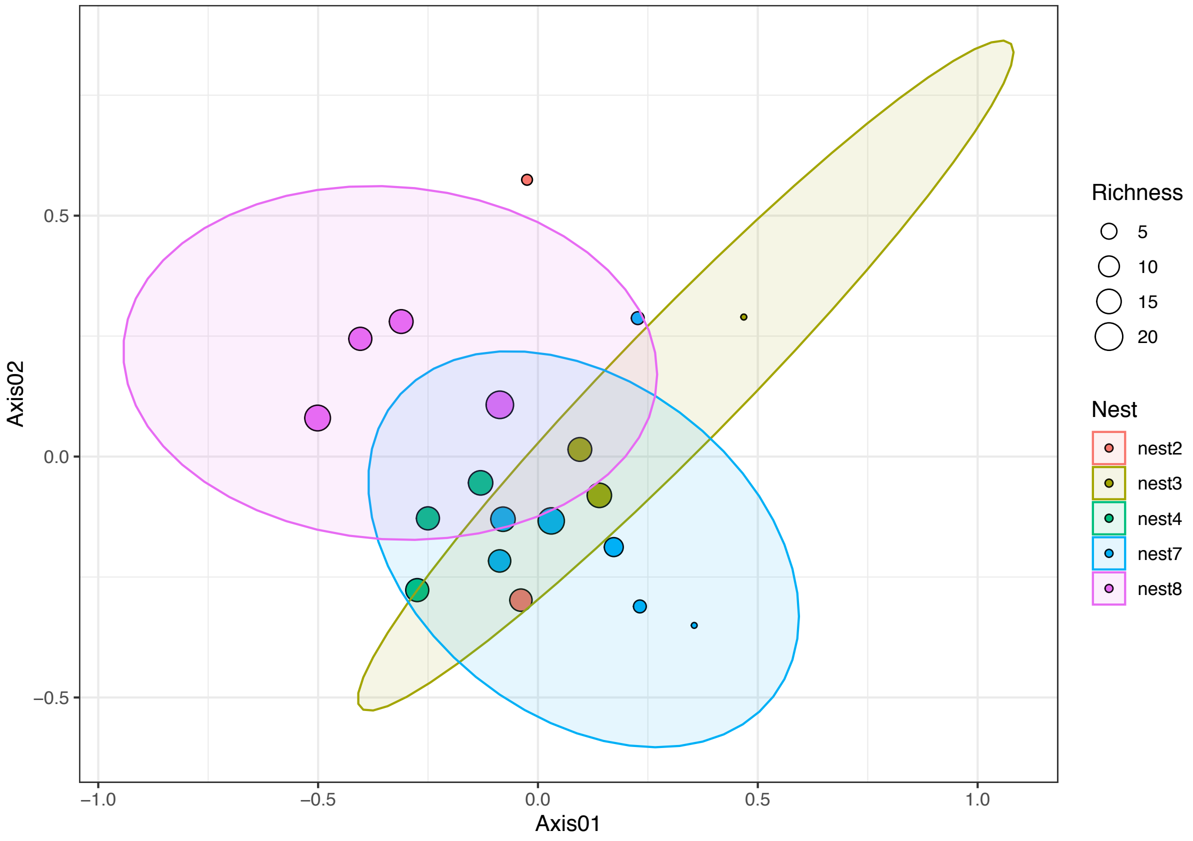

Additional File 7: NMDS analyses of *T. rubida* microbiomes from early instar (L1-L3) individuals found in different *N. albigula* nests found at UADS.
