# Supplementary material for: Ontogeny, species identity, and environment dominate microbiome dynamics in wild populations of kissing bugs (Triatominae)
Source: Microbiome. 2020 Oct 11;8:146. doi: 10.1186/s40168-020-00921-x (PMC7549230; doi:10.1186/s40168-020-00921-x)
Supplement: Supplementary file 9 — Additional File 8: Bartonella phylogenetic analysis of gltA sequences retrieved from T. rubida. [file 40168_2020_921_MOESM8_ESM.pdf]

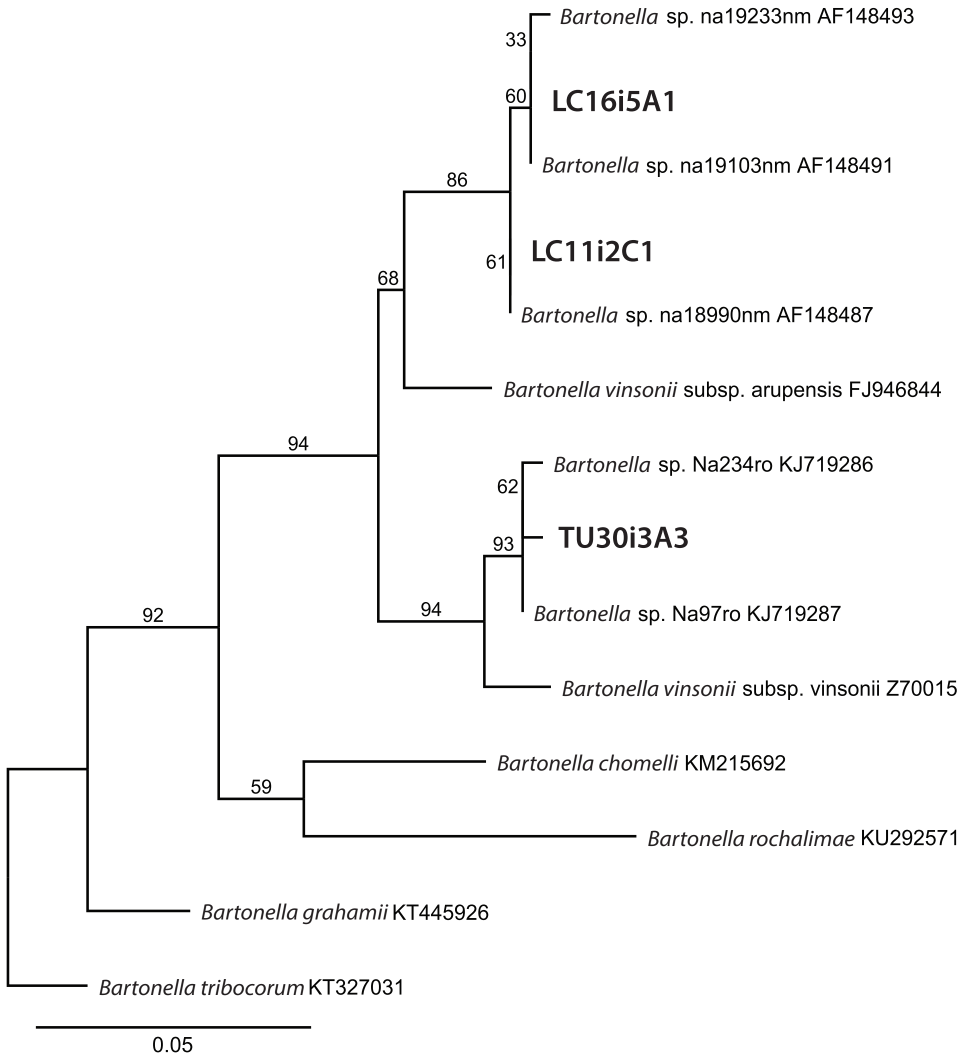

Additional File 8. Maximum-likelihood phylogenetic tree for the partial *gltA* sequence of *Bartonella* spp. Designated samples represent 12 (LC16i5A1), 5 (LC11i2C1) and 1 (TU30i3A3) other sequences retrieved from *T. rubida* individuals in this study. The numbers at the nodes designate bootstrap values.
